# How do selective attentional processes contribute to maintenance and recall in children’s working memory capacity?

**DOI:** 10.3389/fnhum.2014.01011

**Published:** 2014-12-16

**Authors:** Hannah E. Roome, John N. Towse, Chris Jarrold

**Affiliations:** ^1^Department of Psychology, Lancaster UniversityLancaster, UK; ^2^School of Experimental Psychology, University of BristolBristol, UK

**Keywords:** selective attention, working memory capacity, primary memory, secondary memory, dual-component model, presentation modality

## Abstract

The development of working memory capacity is considered from the perspective of the active maintenance of items in primary memory (PM) and a cue-dependent search component, secondary memory (SM). Using free recall, plus a more novel serial interleaved items task, age-related increases in PM estimates were evident in both paradigms. In addition to this, age-related improvements in attentional selectivity were observed, indexed by the recall of target and non-target information respectively. To further characterize PM, presentation modality was varied in the serial interleaved items task (auditory, visual and dual presentation). Developmental differences were found in the effectiveness of presentation formats. Older children’s recall was enhanced by the combination of labeled visual items and enduring auditory information, whilst the same format was detrimental to younger children’s recall of target information. The present results show how estimates of PM and SM in children relate to the development of working memory capacity, but measurement of these constructs in children is not straightforward. Data also points to age-related changes in selective attention, which in turn contributes to children’s ability to process and maintain information in working memory.

## INTRODUCTION

Working memory is frequently described as a memory system responsible for the active maintenance of task-relevant information, alongside other concurrent processing (Baddeley and Hitch, 1974). The enormous interest in, and popularity of, working memory capacity tasks such as reading span (Daneman and Carpenter, 1980) and counting span (Case et al., 1982) partly reflects the way in which these paradigms operationalize this conceptual description. At its core, working memory capacity involves the management of to-be-remembered items on the one hand and concurrent representations on the other. This has generated interest in for example inhibitory processes for the gating of information into the system, the ‘housekeeping’ of information already there (Kane and Engle, 2002; Hasher et al., 2007) and the impact of representational overlap between memory items and processing items (Saito and Miyake, 2004). It is therefore important to understand the attentional mechanisms involved in which items are actively maintained and which are not. In this paper, one key objective is to describe the contribution of attentional selectivity to the development of working memory processes.

The dual-component model (Unsworth and Engle, 2007) provides an influential analysis of working memory capacity in adults, which also draws on ideas relevant to attentional mechanisms. According to this perspective, working memory capacity comprises two memory systems: primary memory (PM) and secondary memory (SM). PM is a flexible memory system that actively maintains a fixed number of memory representations (Waugh and Norman, 1965; Unsworth and Engle, 2007), whilst SM is driven by cue-dependent search processes to recall target-relevant information (see Towse et al., 2008, for one perspective on search mechanisms underpinning working memory recall). Unsworth and Engle (2007) argued that the division of responsibility between these two systems explains individual differences in working memory capacity and how such differences are linked to wider cognition. To explore this dichotomy, Unsworth and colleagues (Unsworth and Engle, 2007; Unsworth et al., 2010, 2011) initially used free recall to obtain estimates of PM and SM. Based upon the serial positions of recalled items, the superior recall of recency items reflected the unloading of items from PM, whilst recall of the primacy or asymptote sections reflected cue-dependent search processes required for retrieval from SM. Yet, to prove that the same mechanisms required in free recall are also required in complex span tasks (a traditional measure of working memory capacity), free recall measures, were shown to load as highly as complex span tasks onto working memory capacity (Engle et al., 1999). From this, the dual-component model (Unsworth and Engle, 2006, 2007; Unsworth et al., 2010, 2011) have delivered an intriguing perspective to working memory, demonstrating how PM and SM use provide unique contributions to working memory capacity.

The use of free recall to obtain estimates of PM and SM has been pivotal to the implementation of the framework to explain *adult* working memory capacity. However, little is known about the developmental performance on free recall paradigms and the emergence of PM and SM capacities through childhood. Accordingly, a second objective is to establish whether the acknowledged change in memory in childhood (Case et al., 1982) is accompanied by developmental increases in PM capacity, SM capacity, or both. There are few published studies directly relevant to the development of PM and SM. One exception is De Alwis et al. (2009), who argued for age-related increases in SM, but not PM. The idea that recency performance does not change with age echoes earlier work by Cole et al. (1971) and Thurm and Glanzer (1971). Yet recent work by Jarrold et al. (in press) has led to an alternative conclusion that developmental increases in PM are observable, after controlling for individual’s order of report. The current work aims to provide further evidence as to whether free recall supports the idea that PM develops with age. Further, presentation rate and list length were varied, thought to affect primacy effects (Murdock, 1962) to explore whether such manipulations affect the relative contributions of PM and SM to output.

Currently, free recall carries a heavy burden in deriving measures of PM and SM, especially as the adult-based algorithm of Tulving and Colotla (1970) for separating these systems is of questionable validity among children. Their method assumes that the lag length between the presentation and recall of items [labeled an intratrial retention interval (ITRI)] specifies which system is used. Items with an ITRI of seven or below are believed to be recalled from PM, whilst items with an ITRI above seven are deemed to be recalled from SM. However, it is unclear whether this cutoff reasonably applies to children, who recall fewer items overall than adults (Jarrold et al., in press). Tulving and Colotla’s (1970) approach also assumes participants consistently begin their response from recency sections of a list. Variation in the recall order affects the recall lags in ways that may not always map straightforwardly onto the proposed partitioning of memory. Therefore, the distribution of ITRI values generated by children are described to help clarify the extent to which they can accurately recall items despite long lags, and quantifying the prevalence of various lag distances in successful recall. This article also reports what children are able to recall, and where children begin their recall. All these measures offer clues as to whether PM and SM, as derived from Tulving and Colotla (1970), represent distinct and coherent constructs.

In order to help clarify the developmental trajectory of PM, an independent paradigm was administered to provide complimentary measures of this construct. The convergent measure of PM, called the serial interleaved items task, is based on a dichotic listening paradigm (Bryden, 1971) recently revived and adapted by Hall et al. (submitted). Bryden (1971) found that adults recall of ‘attended information’ was impaired when delayed through the requirement to report ‘unattended’ items first. This is consistent with the characterization of PM functioning among adults (e.g., Broadbent, 1958; Parkinson, 1974; Martin, 1978). Therefore, a developmentally appropriate implementation of the dichotic task was deployed, largely following Hall et al. (submitted). Instead of using simultaneous presentation of two auditory streams, the two presentation sources were alternated. The two types of stimuli were labeled as focal and non-focal to indicate whether items were designated targets. These terms were preferred over the original labels of ‘attended’ and ‘unattended’ as they refer to experimenter-assigned priorities, but are neutral with respect to attentional control processes. Hall et al. (submitted) argued that performance provides several ‘signatures’ indicative of PM, and reported a developmental increase in PM capacity. Therefore, the current experiment provided the opportunity to replicate and extend this finding that focal recall (an estimate of PM) increases with age.

According to the dual-component model, incoming memory items are actively maintained in PM, however, information that should be ignored and may act as a distraction might potentially displace such memories. To try and capture this account of PM processes, for 80% of trials children were asked to recall focal items in serial order, whilst for the remaining 20% children were instructed to recall non-focal information. By implementing different priorities for the two streams, one can explore the robustness of both the focal items and the non-focal items. PM is thought to retain focal information, but when children are asked to recall lower priority information the involvement of SM may be required. This will be assessed within the inter-relations between free recall measures of PM and SM and focal and non-focal recall.

The relative success at recalling focal and non-focal targets can shed light on the relationship between item management, PM capacity and working memory capacity. Dichotic listening tasks have previously been linked to working memory capacity and attention (e.g., Conway et al., 2001). In order to be successful at such tasks participants have to direct cognition both to form robust memory representations of the focal or to-be-remembered items, and avoid confusing these with non-focal or irrelevant information. Older children are found to be better at selecting just focal items, whilst younger children are less efficient at preventing non-focal intrusions appearing in output (Doyle, 1973; Sexton and Geffen, 1979). This is potentially explained by age-related increases in the ability to focus attention on task–relevant cues, making recall less affected by distracting stimuli (Hagen, 1967). The implementation of an 80–20% split between the recall of focal and non-focal information requires children to filter necessary information in order to be successful at the task, minimizing the number of irrelevant items in working memory (Cowan et al., 2010). Under such conditions, older children should be able to focus attention better on the task at hand and be less affected by distracting stimuli. Overall, using the estimates of PM, SM and working memory capacity, this experiment will assess whether selective attention is relevant to these constructs with respect to the inter-correlations between them.

The interlink between working memory capacity and selective attention in children has been investigated by Cowan et al. (2010, 2011) within the visual domain. As part of assessing visual working memory capacity, the authors used simultaneous (Cowan et al., 2010) and interleaved presentations (Cowan et al., 2011) of attended and unattended stimuli in a visual array task. Cowan et al. (2010, 2011) reported that younger children retained fewer items in working memory, implying that a developmental increase in visual working memory capacity is central to performance. However, age-related differences in the allocation of attention between attended and unattended stimuli was only apparent when the memory load was large relative to working memory capacity. Thus, the developmental changes observed were attributed to an individuals working memory capacity as opposed to their ability to allocate attention effectively. In the study described here, consistent with Cowan et al. (2011) the serial interleaved items task will show an age-related increase in PM capacity, positively linked to increases in working memory capacity. Further, the experiment lends itself to explore how generalizable Cowan’s findings are in a different context, exploring age-related differences in the proportion of focal and non-focal recall, indicative of an effective use of selective attention.

The presentation modality of stimuli on the serial interleaved items task was manipulated, using an auditory, visual, or combined auditory and visual format. This allowed the assessment of whether PM capacity and attentional selectivity is modulated by the nature of representational codes (see Penney, 1989 for a review). In serial recall, auditory presentation produces a stronger recency advantage than visual presentation (Cowan et al., 2002). Further, auditory information has been argued to have privileged or obligatory access to some cognitive systems (Salamé and Baddeley, 1982; McLeod and Posner, 1984). However, within the context of dichotic listening paradigms, Martin (1978) reported similar forgetting of attended visual and auditory items after a delay in adults. Therefore, the current experiment provides the opportunity to explore how different presentation modalities affect the attentional capture and active maintenance of target information in the age range chosen.

In summary, the current experiment compares serial interleaved items and free recall as tasks that draw on PM. Firstly, the proportion of recalled focal and nonfocal items in the interleaved items task provides indpendement indices of PM use, and secondly the ability to selectively attend to target items. Two age groups were chosen: 5–6 year olds and 7–8 year olds, thereby describing early, primary school development, and permitting assessment of whether recall priorities change as selective attention processes mature over this period. Overall, this article considers how an estimate of working memory capacity is composed from a suite of cognitive systems and capacities providing not only theoretical relevance but also practical implications in educational practice.

## MATERIALS AND METHODS

### PARTICIPANTS

Eighty children were recruited from three primary schools in the North-West of England, having obtained parental consent. Children were classified by class into younger (5- to 6-year-olds; *N* = 40, *M* = 6.02 years and months, range: 5.07–6.11, 25 female), and older (7- to 8-year-olds; *N* = 40, *M* = 8.00 years and months, range: 7.02–9.00, 21 female) groups. The sample size was based on previous relevant studies, reporting between 36 and 136 participants. All participants completed all experimental measures within the task, with no exclusions of data. We therefore comply with the recommendations of Simmons et al. (2012), in that “We report how we determined our sample size, all data exclusions (if any), all manipulations, and all measures in the study.”

### MATERIALS

A stimulus pool of 380 words was extracted from the MRC linguistic database (Wilson, 1988). The corpus comprised 236 words used in the free recall experiment; 97 in the interleaved items task and 47 for the listening span task. All stimuli were monosyllabic, concrete nouns, with age of acquisition ratings below 6.2 years. Colored pictures were also presented for the free recall and interleaved items tasks. These were the same visual stimuli used by Hall et al. (submitted) and supplemented with additional items.

### PROCEDURE

Participants took part in the serial interleaved items trials, free recall and listening span tasks in one session lasting approximately 40 min. The order of tasks within the session were counterbalanced across participants. The interleaved items and free recall tasks were programmed using Livecode 5.5 and the listening span task using Psyscript, version 2.3.0 (Slavin, 2003–2014). All experimental events were delivered on a 15-inch screen MacBook laptop, in a quiet, classroom setting.

#### Serial interleaved items task

Children were assigned to one of three presentation conditions: visual (colored illustration only, younger: *N* = 13; older: *N* = 13), auditory (spoken words, younger: *N* = 14; older: *N* = 13) and dual presentation (both presentation forms occurred simultaneously, younger 1: *N* = 13; older: *N* = 14). This task involved two cartoon characters, Spongebob and Patrick, distinguished by two male voices and colored illustrations. Participants were instructed to try and remember Spongebob’s items (focal items) and ignore Patrick’s items (non-focal items), ensuring that the recall of focal information was the focus.

Presentation consistently began with a focal item on the left hand side of the screen with the cartoon Spongebob and then alternated with the non-focal stimuli and cartoon character Patrick on the right hand side. The task included 20 trials with list lengths ranging from three to six items in total. For example, list length three included the alternation of two focal items and one non-focal item. The longest list of six items included the interleaved pattern of three focal and three non-focal items. Stimuli appeared for 1,000 ms with a 250 ms interstimulus interval. The list lengths used were pseudo-randomized and children were not aware of which list length would be presented. After stimulus presentation, 80% of lists were followed with a highlighted red speech bubble appearing above Spongebob on the left hand side of the screen, indicating the recall of focal items. For the remaining 20% of trials the red speech bubble appeared above Patrick on the right hand side of the screen, indicating the recall of non-focal items. The position of the red speech bubble was distributed randomly and therefore participants were unaware of where it was going to appear on each trial. Participants were instructed to use serial recall, thus recalling the focal items in the order in which they were presented.

#### Free recall

List length (8- and 10-items) and presentation rate (1- and 2-s) were manipulated in a blocked format; four blocks comprising six trials. All list items were presented auditorily alongside a colored illustration. Once a list was finished, participants were instructed to recall all the items they could remember in any order.

#### Listening span

The listening span task was adapted from procedures described in Threadgold (2012). Participants listened to sentences whilst trying to remember a set of unrelated words. List length increased sequentially from two to five items, with three trials at each list length, generating 12 trials. There were 42 sentences available, half of which were “silly” (i.e., semantically inappropriate); the other half were not (based on early acquired semantic information, for example “A book is a musical instrument.” in contrast to “I can see with my eyes ”). If children thought the sentence was silly they pressed “Y” on the keyboard; otherwise “N.” Immediately following this response, the unrelated word was presented in a different voice to that of the preceding sentence. At recall, participants were instructed to recall words in serial order.

## RESULTS

### SERIAL INTERLEAVED ITEMS TASK

Three different measures were used to ascertain age- and presentation modality differences within this task. The proportion of recalled focal targets was used as a measure of PM, but also the ability to selectively attend to the target information. This follows Cowan et al. (2011) who divided the capacity of items held in memory into different proportions according to the allocation of attention. The same analysis was also carried out for the trials that participants were instructed to recall non-focal information. Finally, children’s total recall (i.e., the sum of focal and non-focal information), labeled as *k,* was defined as an estimate of the total number of items loaded into working memory per trial (Cowan et al., 2011). Each of these in turn should provide evidence of PM capacity and selective attentional differences in PM capacity and working memory.

#### Focal recall

**FIGURE 1:**
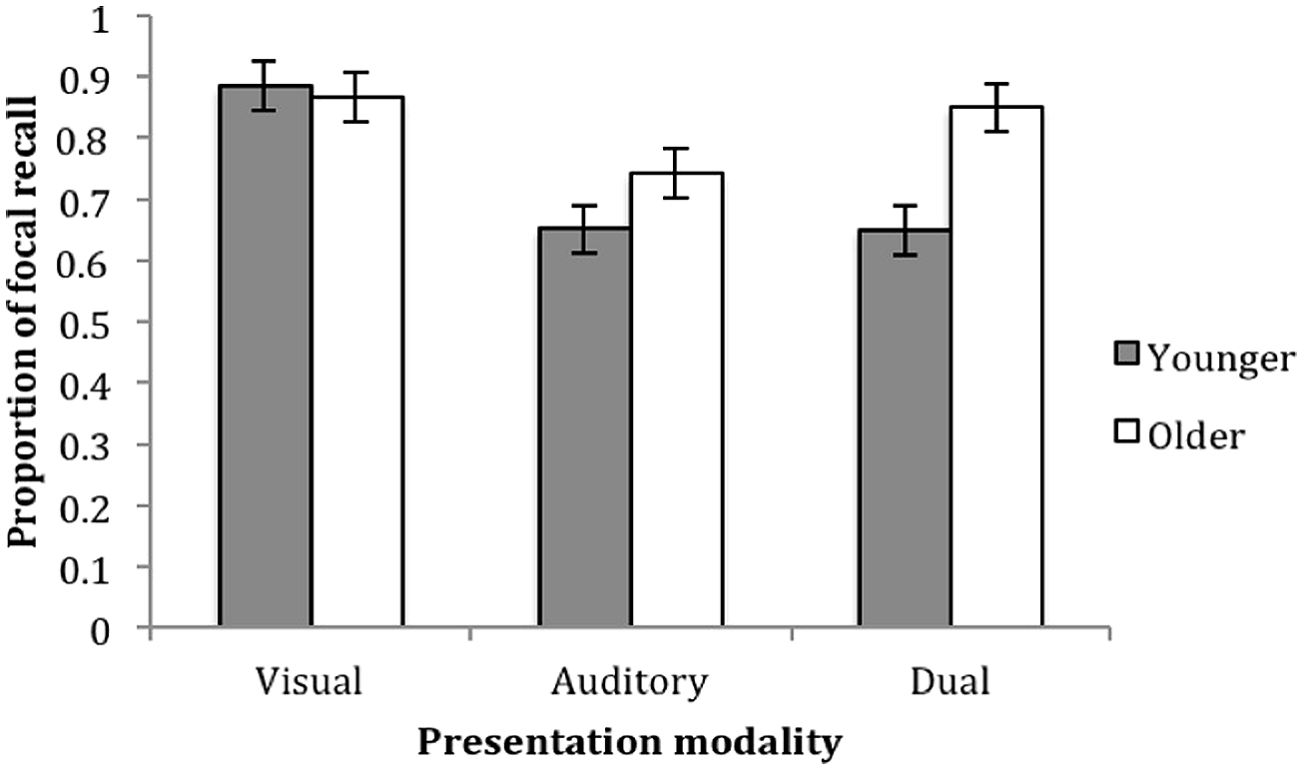
**Proportion of correct focal recall as a function of presentation modality and age.** Error bars represent one SE of the mean.

Analyzing overall proportion of correct focal recall, a 2(age: younger vs. older) × 3(presentation modality: visual vs. auditory vs. dual) ANOVA showed a significant effect of age, *F*(1,79) = 7.561, *p* = 0.007, $\eta_{p}^{2}$ = 0.093 and presentation modality, *F*(2,79) = 10.199, *p* = 0.001, $\eta_{p}^{2}$ = 0.216. Older children recalled a higher proportion of focal items (*M* = 0.819; SE = 0.023) than younger children (*M* = 0.729; SE = 0.023), whilst individuals in the visual condition recalled a higher proportion (*M* = 0.875; SE = 0.029) than both the auditory (*M* = 0.697; SE = 0.028; *p* = 0.001) and dual conditions (*M* = 0.750; SE = 0.028, *p* = 0.011). The interaction between the two variables, *F*(2,79) = 3.641, *p* = 0.031, $\eta_{p}^{2}$ = 0.090 arises because age differences were evident only in the dual condition, *F*(1,26) = 7.735, *p* = 0.010, $\eta_{p}^{2}$ = 0.236 but not the visual: *F*(1,25) = 0.160, *p* = 0.693, $\eta_{p}^{2}$ = 0.007 or auditory: *F*(1,26) = 3.361, *p* = 0.079, $\eta_{p}^{2}$ = 0.119 (**Figure 1**). For additional analyses including list length as a variable please see supplementary materials.

#### Non-focal recall

On those occasions when children were probed for non-focal targets, younger children recalled more than older children (younger: *M* = 0.466; SE = 0.031; older: *M* = 0.328; SE = 0.032), *F*(1,79) = 13.757, *p* = 0.002, $\eta_{p}^{2}$ = 0.157. The dual condition afforded greater non-focal recall, (*M* = 0.531; SE = 0.032), than either the visual, (*M* = 0.274; SE = 0.033), or auditory conditions, (*M* = 0.380; SE = 0.032), *F*(2,79) = 15.644, *p* = 0.001, $\eta_{p}^{2}$ = 0.297. The interaction was marginally significant, *F*(2,79) = 3.016, *p* = 0.055, $\eta_{p}^{2}$ = 0.075, whereby the recall of visual non-focal targets was least accurate and did not differ between age groups, *F*(1,25) = 0.019, *p* = 0.891, $\eta_{p}^{2}$ = 0.001, whilst in both auditory and dual conditions, younger children recalled more items than older children, auditory: *F*(1,26) = 18.545, *p* = 0.001, $\eta_{p}^{2}$ = 0.426; dual: *F*(1,26) = 6.594, *p* = 0.017, $\eta_{p}^{2}$ = 0.209.

#### The use of *k* as a measure of working memory

**FIGURE 2:**
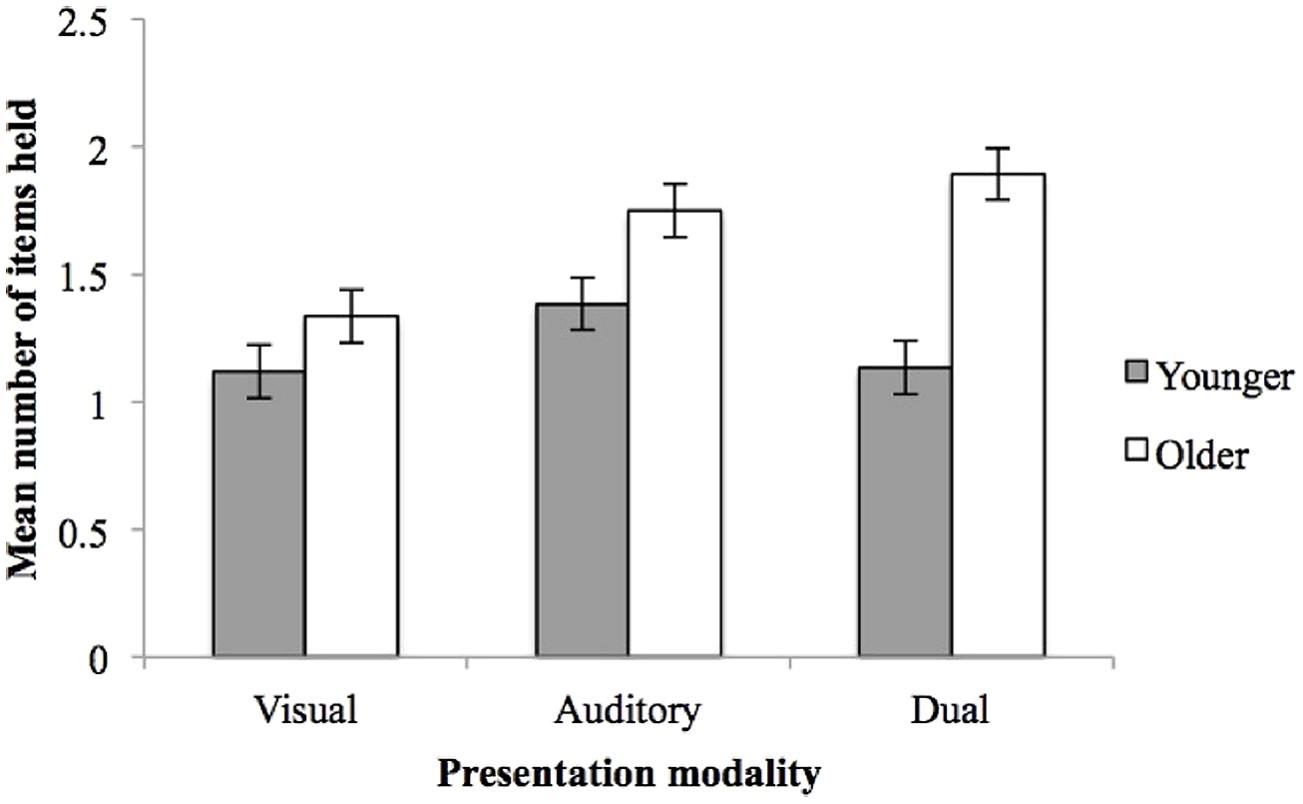
**The mean number of items loaded in working memory (*k*) as a function of presentation modality and age.** Error bars represent one SE of the mean.

The mean number of items in working memory were also analyzed as a function of age and presentation modality. Analysis of variance confirmed older children held more items in working memory than younger children (*M* = 1.660; SE = 0.060 vs. *M* = 1.213; SE = 0.060), *F*(1,79) = 28.091, *p* = 0.001, $\eta_{p}^{2}$ = 0.275. *k* was smallest with visual presentation (*M* = 1.228; SE = 0.074) compared with auditory (*M* = 1.567; SE = 0.073, *p* = 0.006) and dual conditions (*M* = 1.514; SE = 0.073, *p* = 0.015), *F*(2,79) = 6.146, *p* = 0.003, $\eta_{p}^{2}$ = 0.142. A breakdown of the age by presentation interaction, *F*(2,79) = 3.672, *p* = 0.030, $\eta_{p}^{2}$ = 0.090, showed that younger children did not show reliable modality effects, *F*(2,39) = 2.004, *p* = 0.149, $\eta_{p}^{2}$ = 0.098, whilst older children did, *F*(2,39) = 8.278, *p* = 0.001, $\eta_{p}^{2}$ = 0.309, *k* being significantly larger for the dual than the visual condition (*p* = 0.001, **Figure 2**).

### FREE RECALL

Three measures were extracted here; (1) the probability of recall, revealing the serial positions of children’s successful and unsuccessful recall; (2) the probability of first recall, to establish the starting point of children’s recall; and (3) a decomposition of the recall report into PM and SM. Each of these are considered in turn.

#### Probability of recall

**FIGURE 3:**
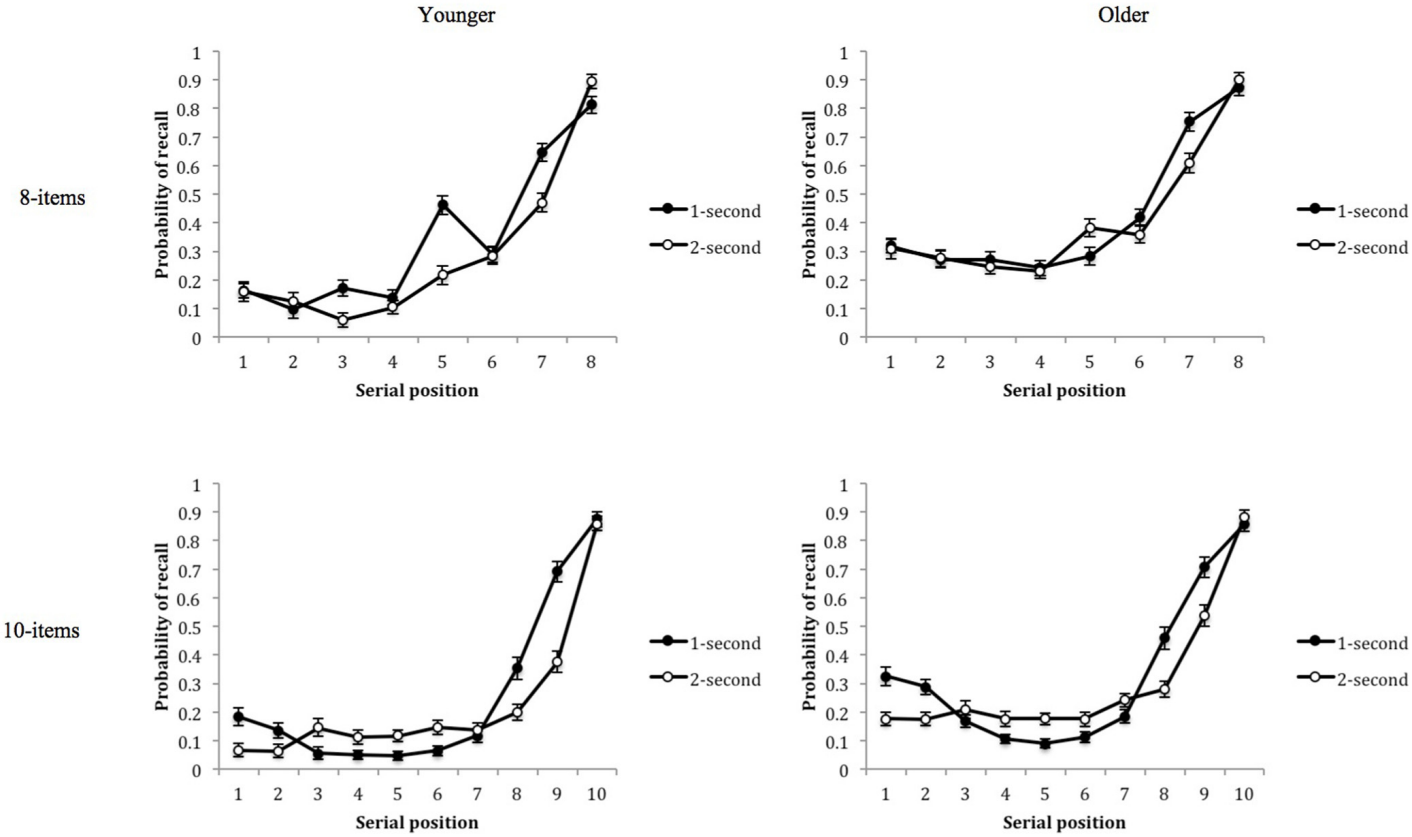
**Probability of recall as a function of serial position, presentation rate and age for 8- and 10-item lists.** Error bars represent one SE of the mean.

Each list length was analyzed separately, investigating the effect of age, presentation rate and serial position (see **Figure 3**). As expected, both analyses showed older children recalled more items than younger children, 8-item lists: *F*(1,78) = 54.520, *p* = 0.001, $\eta_{p}^{2}$ = 0.411; 10-item lists: *F*(1,78) = 44.438, *p* = 0.001, $\eta_{p}^{2}$ = 0.363. Alongside this, there were highly significant main effects of serial position, 8-items: *F*(7,546) = 274.131, *p* = 0.001, $\eta_{p}^{2}$ = 0.778; 10-items: *F*(9,702) = 323.351, *p* = 0.001, $\eta_{p}^{2}$ = 0.806. There were clear recency effects at both list lengths, in which the final items significantly differed from each other, but also all pre-recency items (all *ps* = 0.001). A primacy effect was only established for the 10-item list and was evident only among older children, leading to an interaction between serial position and age, *F*(9,702) = 1.946, *p* = 0.043, $\eta_{p}^{2}$ = 0.024.

Children recalled more items at the faster presentation rate (1- vs. 2-s per item), for both 8- and 10-item lists, *F*(1,78) = 18.200, *p* = 0.001, $\eta_{p}^{2}$ = 0.189 and *F*(1,78) = 17.020, *p* = 0.001, $\eta_{p}^{2}$ = 0.179 respectively. With 8-item lists, the presentation rate effect was only evident in younger children, *F*(1,39) = 21.648, *p* = 0.001, $\eta_{p}^{2}$ = 0.357, thus the significant interaction between presentation rate and age, *F*(1,78) = 5.915, *p* = 0.017, $\eta_{p}^{2}$ = 0.070. In addition, we found significant interactions between serial position and presentation rate, *F*(7,546) = 5.848, *p* = 0.001, $\eta_{p}^{2}$ = 0.070, and a three-way interaction between serial position, presentation rate and age, *F*(7,546) = 5.921, *p* = 0.001, $\eta_{p}^{2}$ = 0.071, reflecting how the effect of presentation rate on the different age groups was portrayed across the eight serial positions. As shown in **Figure 3**, younger children produced higher recall at the 1-s rate across different serial positions [position three: *t*(39) = 4.000, *p* = 0.001, five: *t*(39) = 5.267, *p* = 0.001 and seven: *t*(39) = 3.902, *p* = 0.001], whilst this was only evident at position seven in older children, *t*(39) = 4.451, *p* = 0.001.

The 10-item lists generated a significant interaction between serial position and presentation rate, *F*(9,702) = 23.428, *p* = 0.001, $\eta_{p}^{2}$ = 0.231, with the faster rate producing greater primacy and recency, at positions one, two, eight, and nine [all *ts*(79) > 3.789, *ps* = 0.001]. Yet, the slower presentation rate generated higher recall between middle positions three and six, [all *ts*(79) > -2.552, *ps* < 0.05]. The interaction between presentation rate and age was not significant, *F*(1,78) = 0.260, *p* = 0.611, $\eta_{p}^{2}$ = 0.003, nor was the three-way interaction between serial position, presentation rate and age, *F*(9,702) = 1.430, *p* = 0.171, $\eta_{p}^{2}$ = 0.018.

#### Probability of first recall

**FIGURE 4:**
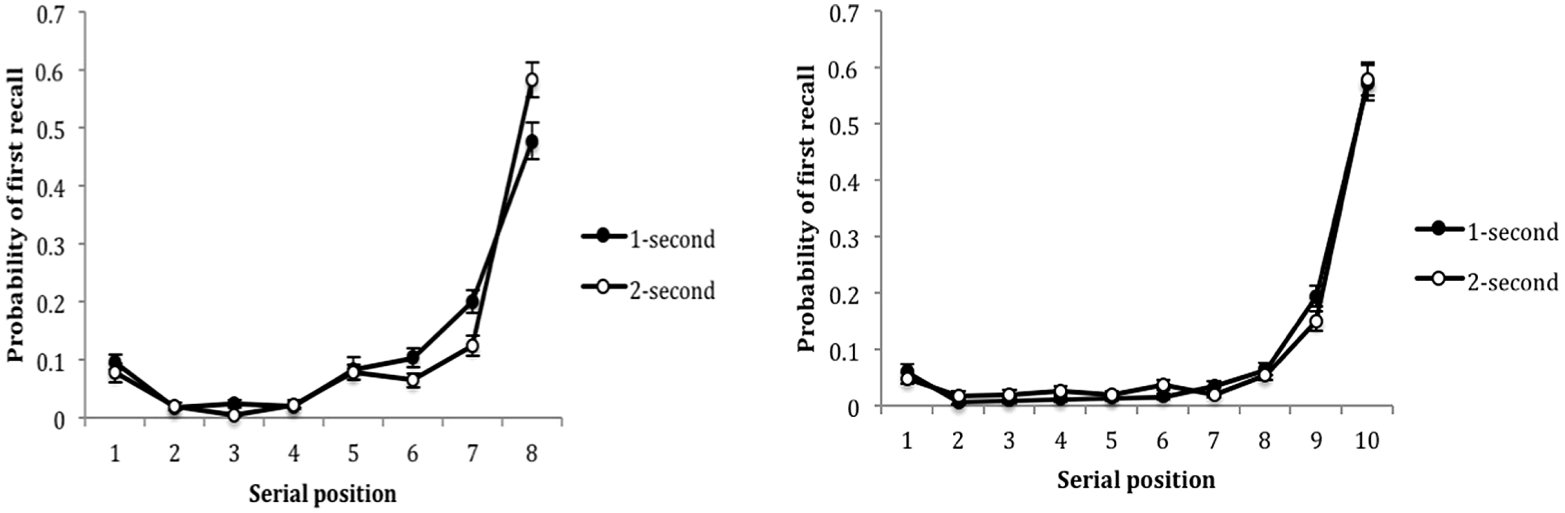
**The probability of first recall as a function of serial position and presentation rate for 8- and 10-item lists.** Error bars represent one SE of the mean.

Where did children begin their recall? For 8-item lists, the last serial position was the most likely entry point for all children’s recall, *F*(7,546) = 158.585, *p* = 0.001, $\eta_{p}^{2}$ = 0.670 (**Figure 4**). This was the case for both age groups as there was no significant age group effect, *F*(1,78) = 0.275 *p* = 0.601, $\eta_{p}^{2}$ = 0.004. The interaction between serial position and presentation rate, *F*(7,546) = 5.690, *p* = 0.001, $\eta_{p}^{2}$ = 0.068, revealed that at the faster presentation rate, children showed a raised probability of beginning their recall at positions six, *t*(79) = 1.966, *p* = 0.050 and seven, *t*(79) = 3.232, *p* = 0.002 in comparison to the slower 2-s rate. However, when making the same comparison, the probability of beginning recall with the last item was higher at the slower rate, *t*(79) = -2.926, *p* = 0.004 (**Figure 4**). The 10-item lists only showed a significant effect of serial position, *F*(9,702) = 232.789, *p* = 0.001, $\eta_{p}^{2}$ = 0.749, whereby the final position was the most likely point for participants to begin their recall, but no effect of age, *F*(1,78) = 1.070, *p* = 0.304, $\eta_{p}^{2}$ = 0.014, and no interaction between serial position and presentation rate, *F*(9,702) = 1.396, *p* = 0.186, $\eta_{p}^{2}$ = 0.018 were evident.

#### PM and SM

Using the original method by Tulving and Colotla (1970), PM and SM estimates were extracted from the free recall method. A 2(list length: 8-items data vs. 10-items) × 2(presentation rate: 1 vs. 2 s) × 2(memory systems: PM vs. SM) × 2(age: younger vs. older) mixed factor ANOVA confirmed older children recalled more items than younger children, *F*(1,78) = 69.050, *p* = 0.001, $\eta_{p}^{2}$ = 0.470, plus greater levels of PM than SM were produced, *F*(1,78) = 2346.401, *p* = 0.001, $\eta_{p}^{2}$ = 0.968. In addition, greater recall was generated at the faster rate, *F*(1,78) = 17.212, *p* = 0.001, $\eta_{p}^{2}$ = 0.181, plus a recall advantage for shorter list lengths, *F*(1,78) = 7.614, *p* = 0.007, $\eta_{p}^{2}$ = 0.089 (see **Table 1**).

**Table 1:** Descriptive statistics of total recall as a function of age, presentation rate, list length and memory systems (one SE of the mean).

	Age		Presentation rate		List length		Memory system	
	Younger	Older	1 s	2 s	8-items	10-items	PM	SM
Mean total recall	1.201 (0.036)	1.630 (0.036)	1.476 (0.027)	1.355 (0.032)	1.446 (0.028)	1.384 (0.029)	2.351 (0.034)	0.480 (0.030)

**FIGURE 5:**
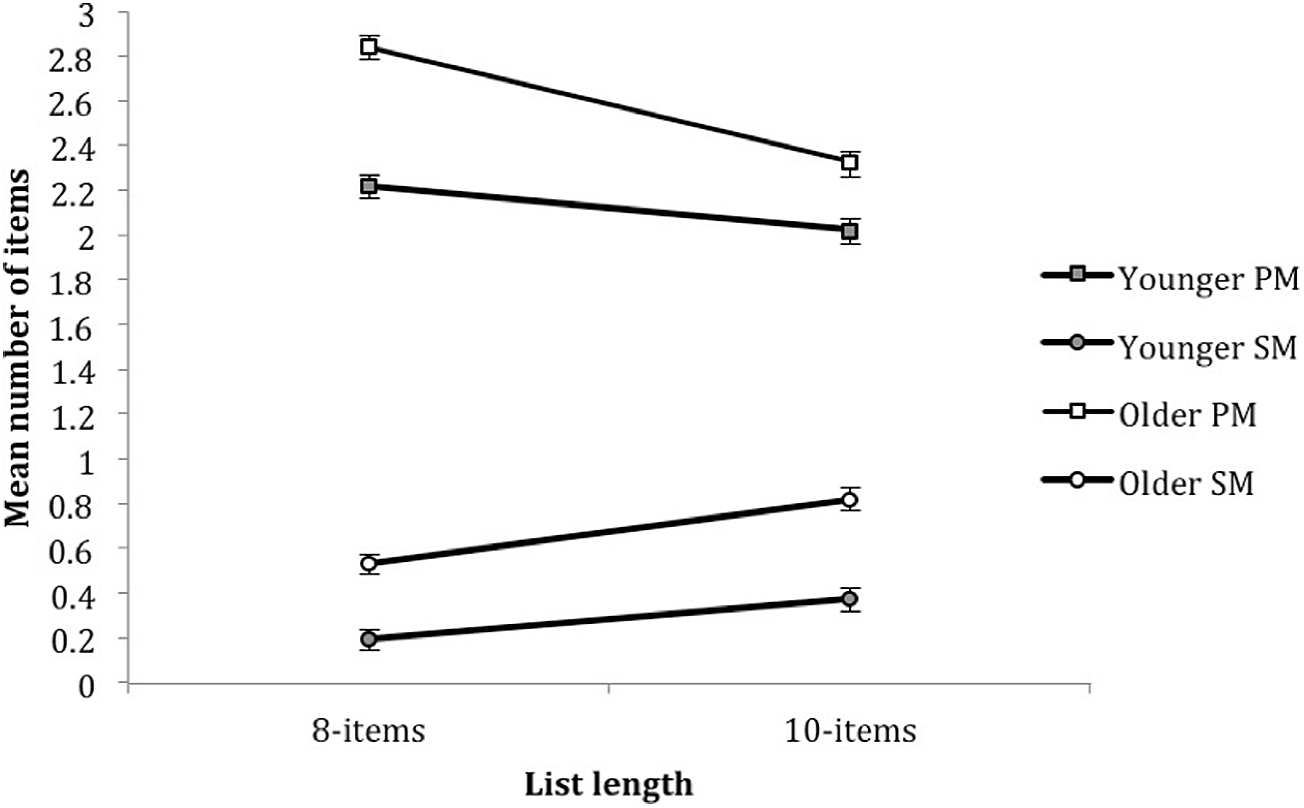
**Mean number of items recalled as a function of list length, memory system and age.** Error bars represent one SE of the mean.

Significant interactions between list length and age, *F*(1,78) = 5.440, *p* = 0.022, $\eta_{p}^{2}$ = ,065; and list length and memory system, *F*(1,78) = 83.425, *p* = 0.001, $\eta_{p}^{2}$ = 0.517, was further qualified by a significant three-way interaction between the three factors, *F*(1,78) = 10.694, *p* = 0.002, $\eta_{p}^{2}$ = 0.121. Both age groups showed a trade-off between PM and SM use. PM use decreased as a function of list length, younger children: *F*(1,158) = 7.477, *p* = 0.007, $\eta_{p}^{2}$ = 0.045; older children: *F*(1,158) = 55.035, *p* = 0.001, $\eta_{p}^{2}$ = 0.258, whilst SM use increased, younger children: *F*(1,158) = 14.245, *p* = 0.001, $\eta_{p}^{2}$ = 0.083; older children: *F*(1,158) = 14.010, *p* = 0.001, $\eta_{p}^{2}$ = 0.081. However, the interaction between list length and age highlighted that the amounted recalled across list lengths did not differ in younger children, *F*(1,39) = 0.077, *p* = 0.783, $\eta_{p}^{2}$ = 0.002, whilst older children recalled more items from the shorter list length, *F*(1,39) = 12.025, *p* = 0.001, $\eta_{p}^{2}$ = 0.236 (**Figure 5**).

**FIGURE 6:**
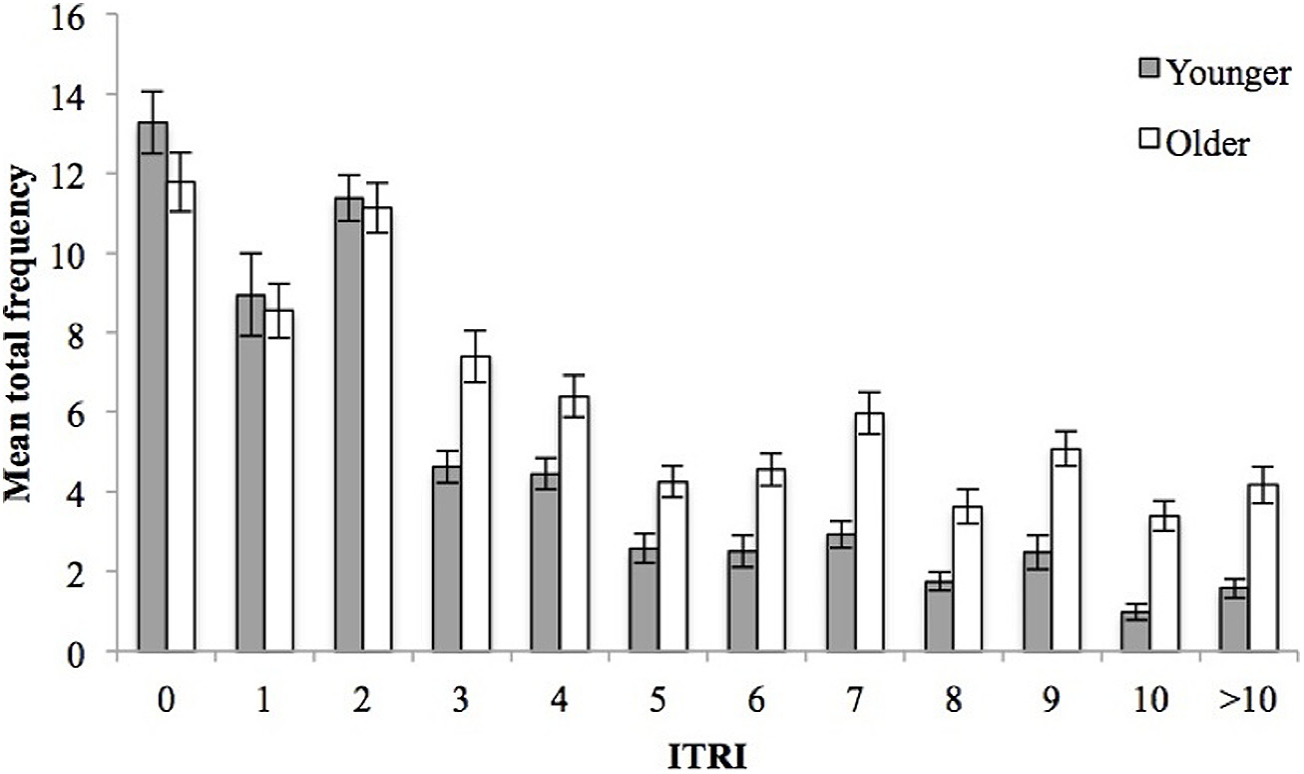
**Frequency of items recalled as a function of intratrial retention interval (ITRI) and age.** Error bars represent one SE of the mean.

The analysis reported thus far is predicated on the assumption that PM and SM are distinguishable by a lag value of ± seven items. It is doubtful that this is appropriate for children, given their pattern of free recall. At the same time, using any other (smaller) threshold for children, without convergent evidence, might be, similarly arbitrary. Therefore, the total frequency of recalled items for each ITRI was calculated to visually depict the recall profile, see **Figure 6**. It is clear that the majority of children based their recall between ITRIs zero and two. The lag profile serves to emphasize how much the recall is based on very short lags. However, these data are not of themselves diagnostic of memory systems, and we do not take them to imply that recall is entirely a reflection of PM.

### THE RELATION BETWEEN SELECTIVE ATTENTION, PM, SM AND WORKING MEMORY CAPACITY

**Table 2** reports the bivariate and partial correlations controlling for age between measures of *k*, focal and non-focal recall taken from the serial interleaved items tasks, working memory capacity (derived from listening span performance) and free recall estimates of PM and SM. There are consistent, significant relationships between *k*, working memory capacity, PM and SM. Therefore, children who recalled more items overall in the interleaved items task showed a higher usage of PM and SM and a higher working memory capacity. Focal recall also significantly related to the measures of working memory capacity, PM and SM, consistent with the view that the ability to select target information is relevant to the listed memory measures. Non-focal recall did not hold significant correlations with any of these variables.

**Table 2:** The correlational analysis between all experimental measures.

	1	2	3	4	5	6
(1) *k*	-	0.813***	-0.047	-0.488***	0.458***	0.534***
(2) Focal recall	0.756***	-	-0.230*	0.523***	0.485***	0.537***
(3) Non-focal recall	0.134	-0.086	-	-0.080	-0.145	-0.223*
(4) WMC	0.335**	0.378***	0.092	-	0.533***	0.624***
(5) PM_fr_	0.239*	0.273*	0.072	0.349**	-	0.544***
(6) SM_fr_	0.350**	0.351**	-0.035	0.481***	0.284*	-

The lower triangle reports the partial correlations controlling for age. *k*, Items loaded in working memory; WMC, working memory capacity; PM_fr_, primary memory estimates from free recall; SM_fr_, secondary memory estimates from free recall. *p < 0.05, **p < 0.01, ***p < 0.001.

## DISCUSSION

An extensive body of research has shown that complex span measures, as indices of working memory capacity, strongly predict complex cognition among children (see Jarrold and Towse, 2006). There have also been many attempts to identify the key components of complex span performance responsible for its psychological profile, in particular the nature of active maintenance (Towse et al., 2007) and the coordination of processing and memorial demands (Jarrold and Bayliss, 2007). The current study enriches such analyses by highlighting the attentional processes that contribute to performance, and distinguishing between highly accessible information (PM) and search processes that operate upon more distributed and diverse representations (SM).

The serial interleaved items task is believed to reflect PM processes (Hall et al., submitted). Older children maintained a higher total number of items in working memory (*k*) as well as a higher proportion of target, focal items, indicating an age-related increase in capacity and PM. We also found that focal recall was maximized and non-focal recall was minimized following visual item presentation. We suggest this may be a labeling effect. Labeling (i.e., vocal naming) only focal items reduced the requirement to filter out irrelevant information, as non-focal items would not be verbally encoded, therefore not interfering with targets. In contrast, auditory presentation produced the lowest proportion of focal recall, a modality believed to have obligatory access to cognitive systems (Salamé and Baddeley, 1982; McLeod and Posner, 1984), even though the *k* measure indicated higher capacity than the visual presentation. Interestingly, the age-related increase in focal recall performance was only evident in the dual condition. The combination of labeled visual items and enduring auditory information enhanced the recall of focal items in older children, whilst younger children’s recall did not change across presentation conditions.

In addition to evidence that PM increased with age, a developing efficiency in selective attention was also observed: an increase in focal recall and decrease in non-focal recall. Differing presentation formats and the nature of representational codes seemed to affect the attentional capture, active maintenance and recall of information. Despite finding low levels of non-focal visual recall in both age groups, younger children’s non-focal recall increased for the other two conditions. This implies that the auditory format hindered the selective maintenance and recall of target information.

The current findings resonate with aspects of Cowan et al. (2011), who found no age differences in attentional allocation with visual working memory capacity. In the current experiment this was also the case, but age differences were obtained in the auditory and dual presented stimuli. Obviously there are key differences between the current experiment and Cowan’s work, in terms of the experimental designs and age groups tested. However, the findings still converge in highlighting the relevance of attention for visual items in working memory. Future work might usefully include the age ranges covered by both experiments, and explore further the modality differences found here. Such work needs to consider whether the distribution of attention across the three presentation formats is equivalent (Cowan et al., 2006), ensuring the visual condition requires equivalent levels of attention as the auditory and dual conditions of the task. This in turn will enable a detailed examination of the specific selective attentional processes involved across the different modalities.

We caution against the conclusion that the serial interleaved items task *solely* relies on PM. One criterion for PM is near-perfect serial output of items (Unsworth and Engle, 2006). This was only evident in the three-item list (mean correct proportion of.93). Only visual recall remained near perfect across list lengths (see Supplementary Materials). Further support for SM contributing to the task was provided by the *k* measure and focal recall correlating with working memory capacity, PM and SM. Together, the results suggest that children’s working memory may face interference from irrelevant, non-focal information, making it harder to recall the target items from PM and potentially forcing the use of cue-dependent search processes in SM. This particular memory system is believed to contribute to performance on complex span tasks as memory items are interleaved with the processing of other information in the environment. The current task also follows this experimental layout, and therefore it may be the case that SM contributes more in longer lists as items are displaced into SM due to the maintenance of new, incoming information in PM.

Children with a higher working memory capacity tended to have a larger *k* score also, and recalled a higher proportion of focal items. This suggests a common role for efficient selective attentional processes in working memory. We were intrigued by the idea of non-focal recall involving retrieval from SM, assuming such items were processed and maintained in the first place. Unfortunately we did not find clear-cut evidence for this view, insofar as non-focal recall did not correlate with any memory measures. However, future research could help verify whether PM and/or SM are involved in the recall of irrelevant information when performing such tasks. The current task only used a small number of trials to assess non-focal recall, and thus it may be the case that this affected the profile of non-focal recall and its relations to the other working memory measures.

In conjunction with the serial interleaved items task, traditional free recall measures were used to illustrate where children begin their recall and what items they were able to recall. The majority of children, regardless of age, began their recall with the final list item, contrasting with reports that show adolescents (Gibson et al., 2010) and adults (Unsworth et al., 2011) more commonly initiate recall with primacy items, This may imply a qualitative change in recall strategy at some point from primary to secondary school. In terms of the serial positions effects, reflecting what items children were able to recall, it was apparent that older children were better at recalling both primacy and recency serial positions than younger children. This is consistent with the recent conclusions of Jarrold et al. (in press), but stands in contrast to previous studies of children’s free recall that suggest age-related increases in primacy but not recency (Cole et al., 1971; Thurm and Glanzer, 1971). The age-related increases in primacy and recency effects also showed up in developmental increases in PM and SM, supporting the work of Jarrold et al. (in press), but conflicting with evidence provided by De Alwis et al. (2009) that PM does not develop with age. One may attribute this conflict in findings to the different methods used to categorize items into the different memory systems. De Alwis et al. (2009) categorized the final four list items as maintenance in PM, and the remaining 10 items retrieved from SM. However, this may be considered an oversimplification, In fact, Jarrold et al. (in press) used the categorization method described by De Alwis et al. (2009) and replicated their results. However, when participants’ order of report was included within the analysis, the age-related increases in PM recall were once again evident. Thus, the data emphasizes the need to use independent measures to derive estimates of PM and SM.

It is important to reflect on the categorization of PM and SM among children from free recall. The original method by Tulving and Colotla (1970) assumes adults levels of PM capacity using a threshold of seven items. Implementing this metric makes it simpler to compare children’s performance here with data from the adult literature. Notwithstanding, if PM capacity is smaller and develops with age, as the interleaved items data suggests, then this method will not truly capture the capacity of either system. SM will be underestimated, and PM exaggerated. **Figure 6** shows that the majority of items recalled were given smaller ITRIs (the highest frequency of ITRIs between zero and two), but the frequency data alone cannot be taken to suggest where to differentiate PM and SM; but to direct to the points of continuity and discontinuity in the profile. In addition, the use of Tulving and Colotla’s (1970) method assumes that recall reports proceed in the same way for all participants (children and adults), which is not borne out by the empirical data (Unsworth et al., 2011), and needs to be considered when debating the validity of this method.

This experiment has provided evidence of the development of PM and SM and the potential application of the dual-component model to children’s memory performance. Correlational evidence linked working memory capacity to both PM and SM. Further, Unsworth and Engle’s (2006; 2007) explanation of complex span as a predominantly SM based task, is also supported by the children’s data. The correlation between working memory capacity (as measured by listening span) and SM was numerically stronger than the correlation between working memory capacity and PM. This suggests that from a young age, the way in which the two systems interact may be similar to adults, although such a conclusion is predicated on the comparability in the algorithms for extracting PM and SM, which have been questioned here (see Jarrold et al., in press).

The current study highlights an increased aged-related ability to distribute attention to target information whilst also ignoring irrelevant information. Younger children are less efficient in their ability to allocate attention, thus they are less able to exclude unnecessary information from working memory. This provides the cognitive underpinnings of the development of PM and SM and how the dual-component model can be applied as an explanation of the development of working memory capacity. The fixed number of memory representations actively maintained in PM and the use of contextual cue-dependent search processes driving SM increase with age throughout childhood. The current article argues though that detailed, independent analyses of the separate components of working memory will help to better model this key cognitive construct. The concepts of PM and SM can help in this, but so can details of processing mechanisms, including selective attention.

## Conflict of Interest Statement

The authors declare that the research was conducted in the absence of any commercial or financial relationships that could be construed as a potential conflict of interest.
