# Supplementary material for: How do selective attentional processes contribute to maintenance and recall in children’s working memory capacity?
Source: Front Hum Neurosci. 2014 Dec 16;8:1011. doi: 10.3389/fnhum.2014.01011 (PMC4267211; doi:10.3389/fnhum.2014.01011)
Supplement: Supplementary file 1 [file Data_Sheet_1.PDF]

21/11/2014

Supplementary Material

Original Research Article

Hannah E. Roome[1],[1],*, John N. Towse[2],[1] Chris Jarrold[3],[2]

[1]Psychology Department, Lancaster University, United Kingdom

[2]School of Experimental Psychology, University of Bristol, United Kingdom

* **Correspondence:** Hannah E. Roome, Psychology Department, Lancaster University, Lancaster, LA1 4YW, United Kingdom, h.roome@lancaster.ac.uk

Supplementary Data

1.1. Proportion of focal recall

The inclusion of list length in the analysis, $F(3,219) = 15.335$, $p = .001$, $\eta_p^2 = .174$, showed the three-item list (the recall of two focal items) produced the highest focal recall (M=.927; SE=.022, all ps = .001) whilst lists four to six did not differ from each other, (four items: M=.668; SE=.032; five items: M=.749; SE=.029; six items: M=.603; SE=.065, all ps > .05). The significant effect of presentation modality, $F(2,73) = 7.354$, $p = .001$, $\eta_p^2 = .168$ displayed the same pattern between the three conditions as previously described, whilst age just missed significance, $F(1,73) = 3.943$, $p = .051$, $\eta_p^2 = .051$. The significant interaction between list length and presentation modality, $F(6,219) = 2.510$, $p = .023$, $\eta_p^2 = .064$ showed no differences between the three conditions at the three-item list, $F(2,79) = .654$, $p = .523$, $\eta_p^2 = .017$, and four-item list, $F(2,79) = 2.074$, $p = .133$, $\eta_p^2 = .051$. From the five-item list onwards, both the auditory and dual conditions focal recall decreased, auditory: $F(3,75) = 26.104$, $p = .001$, $\eta_p^2 = .511$; dual: $F(3,72) = 13.084$, $p = .001$, $\eta_p^2 = .353$, whilst the visual focal recall remained high across list lengths, $F(3,72) = 1.113$, $p = .359$, $\eta_p^2 = .044$ (Supplementary Figure 1).

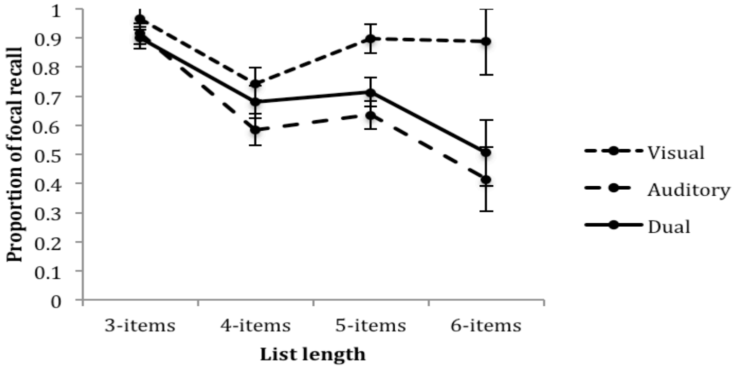

Supplementary Figure 1: Proportion of focal recall as a function of list length and presentation modality. Error bars were one standard error from the mean.

1.2. Proportion of nonfocal recall

List length was also found to impact children's recall of instructed nonfocal information, $F(3,222) = 16.263$, $p = .001$, $\eta_p^2 = .180$ whereby the three-item list generated the highest nonfocal recall (all *ps* =.001) whilst the remaining list lengths showed no differences between each other (all *ps* = 1.000). This was expected as this particular list length required participants to recall one item, whilst the remaining lists required two or three items. The effect of modality, $F(2,74) = 6.618$, $p = .002$, $\eta_p^2 = .152$, highlighted the dual condition to produce a significantly higher level of nonfocal recall than the visual condition ($p = .002$). However, the interaction between these two variables, $F(6,222) = 3.651$, $p = .002$, $\eta_p^2 = .090$, confirmed that the recall of nonfocal visual information remained invariant as a function of list length, $F(3,75) = 1.426$, $p = .242$, $\eta_p^2 = .054$, whilst this effect was prevalent in the auditory, $F(3,78) = 12.260$, $p = .001$, $\eta_p^2 = .320$ and dual conditions, $F(3,78) = 9.353$, $p = .001$, $\eta_p^2 = .265$, whereby recall decreased as the number of nonfocal items to be recalled increased (Supplementary Figure 2). However, by the six-item list there was no systematic difference between all presentation modalities.

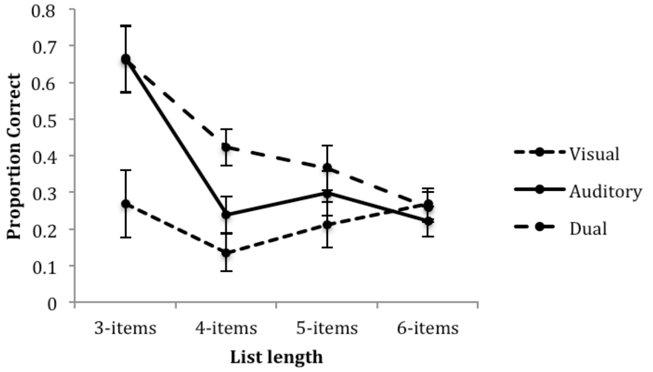

Supplementary Figure 2: Proportion of nonfocal recall as a function of list length and presentation modality. Error bars were one standard error from the mean.

1.3. The use of *k* as a measure of working memory

The significant addition of list length $F(3,222) = 29.683$, $p = .001$, $\eta_p^2 = .286$, showed the three-item list to produce the highest *k* value across all list lengths (*M*=1.792, *SE*=.043, all $ps < .05$). However, the significant interaction between list length and age, $F(3,222) = 6.112$, $p = .001$, $\eta_p^2 = .076$, as illustrated in Supplementary Figure 3, revealed that whilst younger children generated a similar pattern of performance as the main effect just described, $F(3,111) = 22.289$, $p = .001$ $\eta_p^2 = .376$, older children recalled more items on the three- and five-item lists, both of which end in focal items, in comparison to the four- and six-item lists that end in nonfocal items, $F(3,111) = 14.144$, $p = .001$, $\eta_p^2 = .276$.

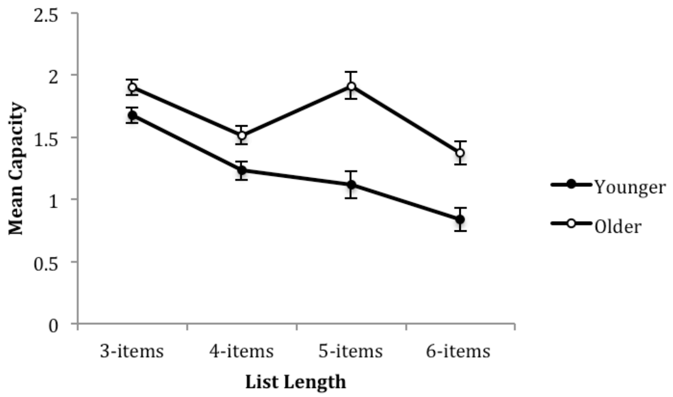

Supplementary Figure 3. The mean number of items loaded in working memory (*k*) as a function of list length and age. Error bars were one standard error from the mean.
